# Surgical treatment of postpartum haemorrhage: national survey of French residents of obstetrics and gynecology

**DOI:** 10.1186/s12884-019-2237-3

**Published:** 2019-03-13

**Authors:** Pierre-Emmanuel Bouet, Hugo Madar, Alizée Froeliger, Hady El Hachem, Elsa Schinkel, Aurélien Mattuizi, Loïc Sentilhes

**Affiliations:** 1 Department of Obstetrics and Gynecology, Montreal University Hospital, Montreal, Canada; 2 Department of Obstetrics and Gynecology, Bordeaux University Hospital, Bordeaux, France; 3 Department of Obstetrics and Gynecology, Clemenceau Medical Center, Beirut, Lebanon; 4 Clinical Research Center, Angers University Hospital, Angers, France

**Keywords:** Survey, Postpartum haemorrhage, Surgery, Residents

## Abstract

**Background:**

Postpartum haemorrhage (PPH) is a major cause of maternal morbidity and one of the leading causes of maternal mortality worldwide. Many medical treatments and interventions are available nowadays, but surgical treatment is sometimes required when less invasive methods are unsuccessful. This study aimed to assess the theoretical and practical knowledge of French residents of Obstetrics and Gynecology concerning the surgical treatment of postpartum haemorrhage.

**Study design:**

We performed a questionnaire study for senior residents of Obstetrics and Gynecology in France (fourth and fifth year of training). An anonymous survey was sent by email. Between December 2013 and April 2014, a total of 370 residents responded.

**Result:**

The response rate was 47.6% (176/370). Only 156 questionnaires were fully completed and included for analysis. In all, 74% (115/156) of residents reported not mastering sufficiently or at all the technique for bilateral ligation of uterine arteries, 79% (123/156) for uterine compression sutures, 95% (148/156) for ligation of the internal iliac arteries, and 78% (122/156) for emergency peripartum hysterectomy. More than half of respondents (55%, 86/156) stated that they had not mastered any of these techniques.

**Conclusion:**

An alarmingly high number of French senior residents in Obstetrics and Gynecology report that they have not acquired the sufficient surgical skills during their training to be able to perform the surgeries required for the management of PPH.

**Electronic supplementary material:**

The online version of this article (10.1186/s12884-019-2237-3) contains supplementary material, which is available to authorized users.

## Background

Postpartum haemorrhage (PPH) is defined as a loss of more than 500 mL of blood within the first 24 h following childbirth and is a major cause of maternal morbidity and the leading cause of maternal mortality [1]. A recent review conducted by the World Health Organisation (WHO) that included data from 115 countries estimated that 27% of maternal deaths worldwide are due to PPH [1]. However, most PPH-related deaths are preventable. Indeed, the French national expert committee recently estimated that more than 80% of mortality cases due to PPH were avoidable [2], a rate reported in many other countries with different demographics [3, 4]. Surgical management is indicated when medical and less invasive approaches fail to control the bleeding, and can be conservative (when it involves simple vascular ligation), or radical (when it involves a hysterectomy). A recent French population based study including more than 140,000 deliveries reported conservative surgical management of PPH in 1.3% of cases and a hysterectomy in 1.1% of cases [5]. The incidence of emergency hysterectomies for PPH in the general population in high resource countries is around 1 per 2000 to 3500 deliveries [5–7]. However, and even though surgical treatment of PPH is rarely needed, it may be the only life saving option in several clinical scenarios, especially after failure of more conservative approaches, such as medical treatment with uterotonic agents, intrauterine tamponade or packing, or uterine artery embolization. Surgical management is even sometimes the only option in certain clinical settings [2].

In France, the law states that all obstetricians/gynecologists should acquire the necessary surgical skills during their training, in order to be able to perform an emergency hysterectomy for PPH [8]. Any failure to adequately perform a surgery for PPH when needed may result in a medical malpractice lawsuit. Therefore, national Obstetrics and Gynecology training programs must ensure that residents are adequately exposed and trained for the surgical management of PPH [9].

However, with the continuous improvement in the medical management of PPH, the number of cases requiring a surgical intervention is low. It is therefore reasonable to suspect that some residents may not be sufficiently trained for such a scenario. The main objective of our study was to assess the theoretical and practical knowledge of senior Obstetrics and Gynecology residents in France concerning the surgical management of PPH. Our secondary objective was to evaluate the surgical management algorithms these residents would implement in their future practice when facing severe cases of PPH (blood loss > 1500 ml) not responding to medical and conservative management.

## Methods

We performed an anonymous questionnaire study, which was approved by the Committee of Ethics and Research in Obstetrics and Gynecology (CEROG-2013-08).

An online survey containing 51 questions was sent by email to 370 post-graduate year 4 (PGY4) and post-graduate year 5 (PGY5) residents of Obstetrics and Gynecology. In France, the national Obstetrics and Gynecology residency training program is a 5-year program, and all residents are registered at the “Association des Gynécologues-Obstétriciens en Formation”, which provided the list of residents and their email addresses. We only included PGY 4 and PGY 5 residents (senior residents) since, according to French legislation they are the only residents allowed to cover maternity wards as senior obstetricians, whether in private or public hospitals [10].

The survey was first piloted in our own department. An email was sent to the 370 eligible residents in December 2013, followed by 2 reminders in February and in April 2014. We collected answers until June 2014, and we only included fully answered questionnaires in the study. A questionnaire was considered eligible only when all questions were answered.

We could not find any validated questionnaire on the subject in the literature, so we designed our survey taking into account all the co-authors’ input. We included binary and open-ended questions, as well as questions with the “other” option in answers in order to elicit alternate responses we could have missed.

The final survey we sent (Additional file 1) had three sections. The first had 12 questions that covered demographic and institutional data and the second had 38 questions about the theoretical and practical knowledge of the residents concerning PPH. The third section contained 3 questions about the surgical management algorithm the participants would apply for severe PPH in a hemodynamically stable patient who wishes to preserve her fertility.

The surgical techniques stated in the survey were: uterine compression sutures (UCS) [11, 12], bilateral uterine artery ligation (UAL) [13], triple uterine artery ligation (TUAL) [14, 15], emergency peripartum hysterectomy (PH) [16], bilateral internal iliac artery ligation (IIAL) and stepwise uterine devascularization (SUD) [17–19].

An email was sent to each participant containing: 1) a cover letter explaining the study, stating that participation was voluntary, unremunerated, and anonymous for all, and indicating the amount of time needed to fill the questionnaire; 2) an internet link that directed the participants to the website (www.limesurvey.com) where the survey was posted. The survey was completed online. Unique identifiers were assigned to each participant’s computer, thus ensuring that they could complete the questionnaire only once. Data were collected via Web-link and email and downloaded to a spreadsheet.

The primary endpoint of our study was the level of knowledge of the residents for each of the surgical techniques included in the survey. This was achieved by self-assessment and the participants had to reply whether they considered their level of expertise as complete, sufficient, insufficient, or absent, for each of the procedures (questions number 18, 24, 30,36, 42 and 48).

Our secondary endpoint was the type of surgery the residents would use as a first, second and third line treatment for PPH (questions 49–51).

Statistical analysis was performed with SPSS 15.0 for Windows (SPSS Inc., Chicago, IL, USA). Descriptive statistics were used to detail demographic characteristics, the theoretical and practical knowledge, and the different management strategies employed by participants.

## Results

The response rate was 47.6% (176/370). Twenty questionnaires were not fully completed and were excluded from the study. One hundred fifty-six questionnaires were thus included in the final analysis.

The participants’ demographic data are presented in Table 1. The median age of participants was 29 years (range: 28–30). They were equally distributed between PGY4 (77) and PGY5 (79) The mean amount of time spent training in surgical departments was 2.4 semesters. The mean number of obstetrical calls was 5.3 per month, and 94% of participants reported having a supervising obstetrician present on site during the calls. Only 14% (22/156) had already covered senior obstetrical calls.

**Table 1:** Characteristics of residents who responded to the survey

	Total *n* = 156
Sex	
• Female	125 (80; 73–86)
• Male	31 (20; 14–27)
Age (years)^a^	29 (28–30)
Semester	8.1 +/− 1.2
• 7th	59 (38; 33–44)
• 8th	18 (11.5; 8–15)
• 9th	61 (39; 36–42)
• 10th	18 (11.5; 8.5–15)
Semesters of surgery residency	2.4 +/− 1.3
Has provided obstetric care alone, without senior obstetrician onsite	
• Yes	22 (14; 9–21)
• No	80 (51; 43–59)
• Not applicable ^b^	54 (35; 27–43)
Number of obstetrical calls/month	5.3 +/− 1.2
Number of obstetrical calls since the beginning of residency	196.3 +/− 84
Considers severe PPH requiring surgical treatment a situation that is:	
• Extremely stressful	48 (31; 24–39)
• Stressful	107 (68.5; 61–76)
• A little stressful	1 (0.5; 0–3.5)
• Absolutely not stressful	0
A senior obstetrician is always onsite when respondent is on-call.	
• Yes	147 (94; 89–97)
• No	9 (6; 3–11)
Considers that he/she knows the department’s protocol for medical management of postpartum haemorrhage:	
• Completely	81 (52; 44–60)
• Sufficiently	68 (43.5; 36–52)
• Insufficiently	7 (4.5; 2–6)
• Not at all	0

Data are expressed as means +/− standard deviations or n (%; 95% confidence intervals)

^a^Median (interquartile range)

^b^Resident in 4th year, during the 7th semester

## 68.6% of residents (107/156) found surgical treatment of PPH to be “stressful” and 30.8% (48/156) found it “extremely stressful”, whereas only one participant considered it not stressful

The primary learning tools reported by residents were textbooks (88%), lectures (53%), the internet (41%), specialized medical journals (31%), and multimedia tools (28%).

There was an important discrepancy between the theoretical and the practical knowledge for most of the techniques. Indeed, for UCS, 99% of respondents (146/156) were satisfied with their theoretical understanding of the technique, but 74% (115/156) reported not mastering it sufficiently or at all. The same was noted for: UAL: 96% (150/156) and 79% (123/156); TUAL 83% (132/156) and 85% (132/156); IIAL 94% (146/156) and 95% (148/156); and PH 96% (150/156) and 78% (122/156). The only surgical method that was not well known to residents, both theoretically and practically, was SUD: 15% (23/156) and 98% (153/156). Finally, 55% (86/156) reported not mastering any of the techniques.

31% of participants (48/156) had already performed UCS with an attending senior colleague, compared to 26% (40/156) for UAL, 19% (29/156) for TUAL, 1% (2/156) for SUD, 12% (19/156) for IIAL and 13% (20/156) for PH (Table 2). On the other hand, 26% (40/156) had never seen nor assisted a UCS, 28% (43/156) a UAL, 46% (72/156) a TUAL, 99% (155/156) a SUD, 44% (69/156) an IIAL and 28% (44/156) a PH. Finally, 7% (11/156) had never seen nor assisted any of the techniques mentioned in a case of severe PPH (Table 2).

**Table 2:** Clinical experience reported by responding residents

	Performed alone ^a^	Performed with the help of a senior obstetrician/gynecologist ^a^	Seen it performed ^a^	Never seen it performed ^a^
Uterine compression sutures	3 (2; 0.5–5.5)	48 (31; 24–39)	75 (48; 40–56)	40 (26; 19–33)
Bilateral ligation of the uterine arteries	1 (0.5; 0–3.5)	40 (26; 19–33)	76 (51; 43–59)	43 (28; 21–35)
Tsirulnikov’s triple ligation	0	29 (19; 13–26)	59 (38; 30–46)	72 (46; 38–52)
Stepwise uterine devascularisation	0	2 (1; 0–5)	1 (0.5; 0–3.5)	155 (99; 96.5–100)
Bilateral ligation of the internal iliac arteries	1 (0.5; 0–3.5)	19 (12; 7.5–18)	69 (44; 36–52)	69 (44; 36–52)
Emergency hysterectomy	1 (0.5; 0–3.5)	20 (13; 8–19)	93 (60; 51.5–67)	44 (28; 21–36)

The data are reported in (%; 95% confidence intervals)

^a^Participating obstetricians reported the number of times they were confronted with each situation for each of the mentioned techniques

When asked about the surgical management of a severe PPH in a young and hemodynamically stable woman who wishes to preserve her fertility, 85 participants (54.5%) responded that their first line treatment would be a distal ligation technique (UAL, TUAL or SUD), whereas 50 participants (32%) chose UCS, 21(13.5%) chose IIAL, and none chose PH. The second line treatment was, in decreasing order, IIAL (42%, 66/156), followed by distal ligation technique (36%, 56/156), UCS (12%, 18/156) and PH (10%, 16/156). Finally, the third line treatment was, in decreasing order, PH (49%, 77/156), IIAL (26%, 40/156), UCS (11%, 17/156), and distal ligation (UAL, TUAL or SUD) (5%, 8/156). All results are summarized in Table 3.

**Table 3:** Techniques that participants would use as first, second, and third line treatment for surgical management of severe PPH in a haemodynamically stable patient

	First line	Third line	Third line
Uterine compression techniques	50 (32; 25–40)	18 (12; 7–18)	17 (11; 6–17)
Distal ligation (of the uterine arteries or Tsirulnikov’s triple ligation or stepwise uterine devascularisation)	85 (54.5; 46–62)	56 (36; 28–44)	8 (5; 2–10)
Bilateral ligation of the internal iliac arteries	21 (13.5; 8–20)	66 (42; 34–50)	40 (26; 19–33)
Emergency hysterectomy	0	16 (10; 6–16)	77 (49; 41–57)
None, a hysterectomy was 12already done		0	16 (10; 6–16)

The data are reported in (%; 95% confidence intervals)

The orders of surgeries most commonly reported by residents were: (1) distal ligation (TUAL or UAL), followed by IIAL, and PH in 29% of cases (45/156); and (2): UCS followed by distal ligation and PH in 20% of cases (31/156).

## Discussion

Our survey shows that most senior Obstetrics and Gynecology residents in France have good theoretical knowledge of the different surgical techniques used in the management of severe PPH. However, an alarmingly high number of these residents -more than half- consider that they have not acquired the sufficient surgical skills during their training to be able to perform these techniques on their own. Moreover, a large proportion reported not being able to perform simple procedures that are considered standard in today’s training programs, such as UAL (79%), TUAL (85%) and UCS (74%).

Several hypotheses can be forwarded to explain the low level of mastery of these surgical methods by senior residents. First of all, the advent of uterine artery embolization (UAE) has significantly reduced the need for these surgeries in the past decade, since it is performed first according to many management algorithms [5, 16, 20]. This may explain, in part, why 25 to 30% of senior Obstetrics and Gynecology residents have never assisted or participated in a UAL, UCS or a PH. The second possibility is that, with the constantly increasing use of the laparoscopic approach in gynaecological surgery over the past 20 years [21], the number of total abdominal hysterectomies done by laparotomy has decreased. Therefore, many residents are not well trained to perform total abdominal hysterectomies in other non-urgent settings, and thus find it hard to perform it in an urgent PPH setting. Indeed, 78% of respondents reported not having the required skills to perform a PH. The third argument is that, in recent years, there has been a trend of overspecialization in the field of Obstetrics and Gynecology, and it is being felt even from the early days of residency training. From our point of view, this might limit the exposure of residents to total abdominal hysterectomies. However, one could argue that overspecialization, as well as the significant improvement in minimally invasive techniques, have considerably improved the quality of care available nowadays. Therefore, the care system has to find new ways and develop new learning tools in order to adjust and keep evolving at the same place. A fourth explanation is the decreasing surgical exposure time of residents since the application of the 2001 French law prohibiting all residents from working on the day following a night call [22]. Finally, since most PPH cases requiring surgical intervention are urgent and life threatening, it is not always possible for the supervising senior obstetrician to take the time to train, guide or assist a junior resident who can be overwhelmed by the situation.

All the aforementioned factors are directly affecting the quality of the current Obstetrics and Gynecology residency training program in France. The issue could be further compounded in the near future if some proposed reforms are applied. These reforms aim at shortening the length of the training program (4 years instead of 5) while guiding residents towards subspecialisation in the field early on during training [23]. Academic professors and physicians in charge of training should be aware of the current difficulties and could develop alternate teaching strategies, such as video teaching sessions [24], specialized books and publications [25, 26], instructional charts, diagrams and iconography [27, 28], and masterclasses and workshops with simulation and hands-on training [29–33]. Such auxiliary educational methods could help alleviate the lack of surgical exposure and play an important part in training residents to deal with cases of severe PPH.

Most residents in our survey preferred the distal ligation (54.4%) and the uterine compression technique (32%) as a first line treatment, rather than IIAL (13.5%). This is in line with several reports in the literature stating that uterine artery ligation and uterine compression are more efficient than IIAL for the control of PPH [16, 27]. Moreover, according to different reports, the mean success rate of IIAL seems to be 69% (39–100%), compared to 93% for uterine artery ligation and 83% for uterine compression [34, 35]. However, due to the lack of direct comparative studies between the different techniques, most international societies refrain from recommending one procedure over the other in their guidelines for the management of PPH [2, 28, 36–38]. Taking into account that more than half of participants (55%) consider they do not master any of the surgical techniques for management of PPH, and that IIAL is associated with a longer learning curve and a higher morbidity [31, 35], we believe it would be more useful to concentrate the efforts on teaching residents the other techniques, such as UAL, UCS and PH, even if the debate on efficacy remains open.

The main limitation of our study is that it is based on a self-assessment questionnaire. Moreover, the response rate was low, but it is comparable to rates reported in similar studies in the literature [39]. However, and to the best of our knowledge, this is the first survey study in the literature assessing the theoretical and practical knowledge of Obstetrics and Gynecology residents concerning the surgical management of PPH. We believe it can be helpful for evaluating and improving training programs in Obstetrics and Gynecology.

## Conclusions

In conclusion, our survey study showed that 55% of French senior residents of Obstetrics and Gynecology consider that they do not master the surgical techniques required for the management of PPH, and that 78% do not have the necessary skills to perform an emergency peripartum hysterectomy. We believe these numbers should serve as an alert that major work is needed in order to improve the training program regarding the surgical management of PPH in France. The various conservative surgical methods have a 70–90% efficiency rate [19, 35] and allow for preservation of future fertility for young patients with PPH, whereas peripartum hysterectomy remains the ultimate life-saving treatment in case of failure of the medical or conservative surgical methods. PPH is the leading cause of maternal mortality, and it is essential that future obstetricians be well trained to surgically manage it.

## Additional file

Additional file 1: Questionnaire about the surgical management of severe postpartum haemorrhage. (DOCX 22 kb)

## Abbreviations

IIAL: Bilateral internal iliac artery ligation
PH: Emergency peripartum hysterectomy
PPH: Postpartum haemorrhage
SUD: Stepwise uterine devascularisation
TUAL: Triple uterine artery ligation
UAL: Bilateral uterine artery ligation
UCS: Uterine compression sutures
