# Supplementary material for: Surgical treatment of postpartum haemorrhage: national survey of French residents of obstetrics and gynecology
Source: BMC Pregnancy Childbirth. 2019 Mar 13;19:91. doi: 10.1186/s12884-019-2237-3 (PMC6415489; doi:10.1186/s12884-019-2237-3)
Supplement: Supplementary file 1 — Questionnaire about the surgical management of severe postpartum haemorrhage. (DOCX 22 kb) [file 12884_2019_2237_MOESM1_ESM.docx]

**Additional file 1. Questionnaire about the surgical management of severe postpartum haemorrhage.**

1 – What is your sex?

- Male
- Female

2 – How old are you?

3 – What semester of residency are you in?

4 - How many semesters of surgery residency have you done?

5 – If you have completed 7 full semesters, are you sometimes responsible for delivery-room coverage?

- Yes
- No
- Not applicable

6 - How many times would you say you are on-call onsite each month, on average?

7 – How many times would you say you have been on-call onsite in the maternity ward since the beginning of your residentcy?

8 – Is there routinely a fully qualified gynaecologist-obstetrician present in the maternity ward and reachable at all times while you are on call?

- Yes
- No

9 – Do you know the protocol for medical management of postpartum haemorrhage in the delivery room

- completely?
- sufficiently?
- insufficiently?
- not at all?

10 – What descriptive tools did you use to learn the different techniques?

Choose all the responses that apply to you:

- None ("on-the-job training")
- Obstetrics textbook
- Internet
- CD-ROM/DVD
- Journals
- Lecture courses
- Other:

11 – Do you think that it is appropriate to ligate the suspensory ligaments of the ovary to control PPH? Please choose only one answer.

- Yes
- No
- Prefer not to say

12 – Do you consider severe PPH requiring surgical treatment a situation that is in principle

- extremely stressful?
- stressful?
- a little stressful?
- absolutely not stressful?

13 –Do you know the theory of uterine compression suture techniques (B-Lynch or Cho)?

- Yes
- No

14 - In practice, have you already (choose all the responses that apply to you)

- performed it alone?
- performed it with help from a fully qualified specialist?
- seen it performed?
- never seen it performed?

15 – How many times have you performed it alone? (answer only if you answered question 14: "performed it alone").

16 - How many times have you done it with help from a qualified specialist? (answer only if you answered question 14: "performed it with help from a qualified specialist")

17 – How many times have you seen it performed? (answer only if you answered question 14: "seen it performed")

18 – Do you consider that you have mastered this surgical technique

- completely?
- sufficiently?
- insufficiently?
- not at all?

19 – Do you know the theory of the surgical technique for bilateral ligation of the uterine arteries?

- Yes
- No

20 - In practice, have you (choose all the responses that apply to you)

- performed it alone?
- performed it with help from a fully qualified specialist?
- seen it performed?
- never seen it performed?

21 – How many times have you performed it alone? (answer only if you answered question 20: "performed it alone)

22 - How many times have you done it with help from a qualified specialist? (answer only if you answered question 20: "performed it with help from a qualified specialist)

23 – How many times have you seen it performed ? (answer only if you answered question 20: "seen it performed)

24 – Do you consider that you have mastered this surgical technique

- completely?
- sufficiently?
- insufficiently?
- not at all?

25 – Do you know the theory of Tsirulnikov's triple ligation technique?

- Yes
- No

26 - In practice, have you (choose all the responses that apply to you)

- performed it alone?
- performed it with help from a fully qualified specialist?
- seen it performed?
- never seen it performed?

27 – How many times have you performed it alone? (answer only if you answered question 26: "performed it alone)

28 - How many times have you done it with help from a qualified specialist? (answer only if you answered question 26: "performed it with help from a qualified specialist)

29 – How many times have you seen it performed? (answer only if you answered question 26: "seen it performed)

30 – Do you consider that you have mastered this surgical technique

- completely?
- sufficiently?
- insufficiently?
- not at all?

31 – Do you know the theory of the stepwise uterine devascularisation technique?

- Yes
- No

32 - In practice, have you (choose all the responses that apply to you)

- performed it alone?
- performed it with help from a fully qualified specialist?
- seen it performed?
- never seen it performed?

33 – How many times have you performed it alone? (answer only if you answered question 32: "performed it alone)

34 - How many times have you done it with help from a qualified specialist? (answer only if you answered question 32: "performed it with help from a qualified specialist)

35 – How many times have you seen it performed? (answer only if you answered question 32: "seen it performed)

36 – Do you consider that you have mastered this surgical technique

- completely?
- sufficiently?
- insufficiently?
- not at all?

37 – Do you know the theory of the surgical technique of ligation of the uterine arteries?

- Yes
- No

38 - In practice, have you (choose all the responses that apply to you)

- performed it alone?
- performed it with help from a fully qualified specialist?
- seen it performed?
- never seen it performed?

39 – How many times have you performed it alone? (answer only if you answered question 38: "performed it alone)

40 - How many times have you done it with help from a fully qualified specialist? (answer only if you answered question 38: "performed it with help from a qualified specialist)

41 – How many times have you seen it performed? (answer only if you answered question 38: "seen it performed)

42 – Do you consider that you have mastered this surgical technique

- completely?
- sufficiently?
- insufficiently?
- not at all?

43 – Do you know the theory of the surgical technique for hysterectomies?

- Yes
- No

44 - In practice, have you (choose all the responses that apply to you)

- performed it alone?
- performed it with help from a qualified specialist?
- seen it performed?
- never seen it performed?

45 – How many times have you performed it alone? (answer only if you answered question 44: "performed it alone")

46 - How many times have you done it with help from a fully qualified specialist? (answer only if you answered question 44: "performed it with help from a qualified specialist")

47 – How many times have you seen it performed? (answer only if you answered question 44: "seen it performed")

48 – Do you consider that you have mastered this surgical technique

- completely?
- sufficiently?
- insufficiently?
- not at all?

49 – In practice, what surgical technique would you use in first-line treatment? Consider the situation of a young woman, haemodynamically stable, desirous of more children, managed by a multidisciplinary team (surgeon and anaesthesiologist). Please choose only one answer.

- Ligation of the uterine arteries or Tsirulnikov triple ligation or stepwise uterine devascularisation.
- Ligation of the internal iliac arteries.
- Uterine compression suture.
- Hysterectomy.

50 – If that failed, what surgical technique would you use as a second line treatment? Consider the situation of a young woman, haemodynamically stable, desirous of more children, handled by a multidisciplinary team (surgeon and anaesthesiologist)

- Ligation of the uterine arteries or Tsirulnikov triple ligation or stepwise uterine devascularisation.
- Ligation of the internal iliac arteries.
- Uterine compression suture.
- Hysterectomy.
- None, a hysterectomy was already done.

51 – If that failed, what surgical technique would you use as a third-line treatment? Consider the situation of a young woman, haemodynamically stable, desirous of more children, handled by a multidisciplinary team (surgeon and anaesthesiologist)

- Ligation of the uterine arteries or Tsirulnikov triple ligation or stepwise uterine devascularisation.
- Ligation of the internal iliac arteries.
- Uterine compression suture.
- Hysterectomy.
- None, a hysterectomy was already done.
